# Challenges in the Physical Characterization of Lipid Nanoparticles

**DOI:** 10.3390/pharmaceutics13040549

**Published:** 2021-04-14

**Authors:** Supandeep Singh Hallan, Maddalena Sguizzato, Elisabetta Esposito, Rita Cortesi

**Affiliations:** 1 Department of Chemical, Pharmaceutical and Agricultural Sciences, University of Ferrara, I-44121 Ferrara, Italy; hllsnd@unife.it (S.S.H.); sgzmdl@unife.it (M.S.); ese@unife.it (E.E.); 2 Biotechnology Interuniversity Consortium (C.I.B.), Ferrara Section, University of Ferrara, I-44121 Ferrara, Italy

**Keywords:** nanoparticles, solid lipid nanoparticles, gels, ethosomes, liposomes, nanostructured lipid carriers, cubosomes, nanotechnology, novel drug delivery system

## Abstract

Nano-sized drug transporters have become an efficient approach with considerable commercial values. Nanomedicine is not only limited to drug delivery by means of different administration routes, such as intravenous, oral, transdermal, nasal, pulmonary, and more, but also has applications in a multitude of areas, such as a vaccine, antibacterial, diagnostics and imaging, and gene delivery. This review will focus on lipid nanosystems with a wide range of applications, taking into consideration their composition, properties, and physical parameters. However, designing suitable protocol for the physical evaluation of nanoparticles is still conflicting. The main obstacle is concerning the sensitivity, reproducibility, and reliability of the adopted methodology. Some important techniques are compared and discussed in this report. Particularly, a comparison between different techniques involved in (a) the morphologic characterization, such as Cryo-TEM, SEM, and X-ray; (b) the size measurement, such as dynamic light scattering, sedimentation field flow fractionation, and optical microscopy; and (c) surface properties, namely zeta potential measurement, is described. In addition, an amperometric tool in order to investigate antioxidant activity and the response of nanomaterials towards the skin membrane has been presented.

## 1. Introduction

Researchers worldwide are working to protect the whole humankind from numerous diseases by designing novel strategies or by improving the existing medicine systems to enrich patient safety and compliance. A drug carrier plays a very essential role in accomplishing good therapy. Substantial development has taken place to combat a number of diseases using different drug cargos, which maintain the concentration, time, and releasing rate of therapeutically active molecules at targeting sites. The main motive of drug delivery systems is to distribute the active moiety to a targeted site within a suitable time window along with a higher concentration in the infected sites and as low as possible in the healthy tissues [1,2,3]. The established and commercially available conventional drug transport systems can increase plasma drug concentration to maintain a therapeutic window for a definite time, and then start declining to an ineffective level followed by the same event on the second administration. However, the achievement of adequate drug concentration for a consistent time during disease state is the major failure in drug therapy. Moreover, conventional drug delivery systems cannot meet certain requirements such as targeted drug delivery, bio-distribution, controlled release, enhanced bioavailability, and mean residence time [3,4]. The design of a suitable drug transporter with the capability of maintaining drug concentration efficiently for a required duration at the desired site without affecting normal cell and body organs is much needed. Nano transporters hold the potential of precise drug delivery through various administration routes, providing benefits including (i) cargo protection and its accumulation at the target site, (ii) improved pharmacokinetics and bio-distribution, reducing dose frequency, (iii) enriched drug transport across biological membranes, and (iv) prolonged or controlled drug release. Thus nanocarriers excellently drive the drug to its target by balancing safety and efficacy [5,6]. Over the past three decades, nanotechnology has been explored a lot by researchers worldwide for biomedical applications. Nanomedicine is not only limited to drug delivery by means of different administration routes such as intravenous, oral, transdermal, nasal, pulmonary, and more, but also has applications in a multitude of areas such as a vaccine, antibacterial, diagnostics and imaging, and gene delivery.

This review will focus on the development of various lipid-based nanosystems with a wide range of applications, but most importantly, on the advancements and challenges of physical characterization techniques and some other techniques that might be supportive in the shift of lab to industrial scale production.

## 2. Nanoscale Carriers

Based on their composition and properties, nanoscale carriers can be divided into two major categories, namely: organic nanoparticles, such as polymer-based particles [7,8], micelles [9], liposomes [10,11,12,13,14,15], ethosomes [16], solid lipid nanoparticles (SLN) [17], nanostructured lipid carriers (NLC) [18], and dendrimers [19]; and inorganic nanoparticles, such as carbon nanotubes [20], graphene nanomaterials [21], gold nanoparticles [22], magnetic nanoparticles [23], and quantum dots [24]. Moreover, their use is proposed for many applications such as diagnostics and therapy.

### 2.1. Drug Delivery

In the field of drug delivery, lipid-based nanosystems have gained a lot of interest thanks to the biocompatibility of the components used in their formulation and to the large therapeutic application and administration routes involved.

Some of the widely investigated lipid-based drug delivery systems along with their physicochemical properties, characterization, and applications have been discussed in this section. Summarized in Table 1 are the principal methods of lipid-based nanoparticle production, together with their advantages and limitations.

#### 2.1.1. Liposomes

Liposomes are the very first successful product of nanotechnology that have been launched into the market for clinical applications. Liposomes are phospholipid-containing vesicles widely accepted for various biomedical applications. Liposomes can be classified into many types depending upon the lipid composition, charge on the surface, diameter, and fabrication process. Phospholipid composition makes them free-moving entities across the biological barriers within the body, hence they are biocompatible [40]. Liposomes have been introduced to research along with various advantages as a successful biomimetic approach. They can be modified to achieve certain aims such as loading of the drug and its target-specific precise release by avoiding rapid clearance [41,42].

Being foreign entities, liposomes are usually cleared out immediately, so a suitable coating material, such as synthetic phospholipids, polyethylene glycol (PEG), or chitin derivatives, is required. Much explored is the ability of PEGylation to escape liposomes from uptake by macrophages to enhance circulation time. Furthermore, the biodistribution of PEGylated liposomes has been adopted successfully in the production of commercially available doxorubicin-loaded liposomes such as Doxil (Janssen Biotech, Inc., Horsham, PA, USA) or Caelyx (Schering- Plough Corporation, Kenilworth, NJ, USA) for the treatment of solid tumors [43]. After administration, liposomes head towards the extravasation into tumor interstitium followed by the uptake via tumor-associated macrophages (TAM). Finally, the intracellular trafficking of liposomes either with endosomes or lysosomes results in the release of drug in the cytosol and infiltration to the nucleo-cytoplasmic membrane with possible DNA destruction [44]. TAM is a contributing factor for tumor growth that alters the tumor microenvironment to diminish growth barriers to protect cancer cells from targeted immune responses. Liposomes can intrude these biological interactions within tumors by altering TAM phenotypes via polarization. This “macrophage polarization” can switch this growth-promoting phenotype to the cancer cell killing phenotype [10]. Particularly, in the case of liposomes, in spite of differences on chemical composition, many groups observed M2-like polarization in macrophages isolated from liposome-treated mice [10,45].

Additionally, liposomes responsive to external stimuli, such as temperature and pH, can be functionalized in order to increase cell permeability. In terms of mild hyperthermia, the vascular permeability of cancer cells will significantly increase the mean residence time of liposomes. Hence, the triggered release of anti-cancer agents on the target site can ensure the effectiveness of this approach [11].

Concerning the biodistribution of liposomes, an approach has been established [12] where liposomes can release drugs, peptides, nucleic acids, or markers directly in the endoplasmic reticulum (ER) membrane in 30 min after entering into cells. In this approach, the composition of the liposomes is very important, because the lipid used should resemble the ER membrane and perform endocytosis into target cells. The membrane fusibility of liposomes is quite challenging because of the complexity of the natural biomembrane and glycoproteins. However, Chen-Yen Wang and colleagues [13] described in their study that liposomal fusion with the membrane could be obtained in the presence of polyhistidine under acidic conditions only. Moreover, the surface charge of liposomes represents an important challenge to increase the interaction with biological membranes. In this regard, negatively-charged liposomes (with the anionic lipid phosphatidylserine) have shown the highest fusion [13]. Furthermore, a study has been conducted to assess the electrostatic adhesiveness of charged liposomes to healthy and colitis-induced intestinal epithelium. Anionic liposomes over cationic and neutral liposomes have presented better results. Additionally, triggered drug release by disease-associated enzymes is another approach to activate prodrugs [46].

#### 2.1.2. Ethosomes

In the 2000s, modification in the production of liposomes with ethanol fraction has emerged as the most preferable approach towards effective transdermal delivery. This second generation of liposomes, along with flexible phospholipid vesicles, is known as ethosomes. The intactness of organized vesicles with 50% of ethanol and the fluidic state of a phospholipid membrane (elastic nature) due to the lowering of transition temperature by ethanol has been verified by ^31^P NMR and differential scanning calorimetry analysis [47,48]. For comparison, both the ethosomes and elastic liposomes have been loaded with a hepatitis B surface antigen for transcutaneous delivery. Both these formulations have shown the ability to stimulate T helper response after uptake by dendritic cells recorded as a protective response against an immune response. However, among both types of carrier systems, ethosomes have been reported for higher internalization and immunogenicity [49].

Additionally, a recent study regarding the development of ethosomes for percutaneous delivery of Coenzyme Q10 revealed that 90% of the drug remained associated with multi-lamellar ethosomes, possibly because of the high interaction of a drug with ethanol and phospholipids. All results from this study reflect that ethosomes loaded with Coenzyme Q10 are very promising in the treatment of oxidative stress [50]. Ethosomes accomplishment is related to the combined functioning of ethanol, vesicle, and skin lipids. The ethanol effect generates the interaction between ethanol and the polar head region of *stratum corneum* (SC) lipids that reduce their transition temperature, ultimately leading to the state of fluidity and decreased density of the lipid multilayer. Secondly, the ethosomes effect includes the fusion of ethosomes with skin lipids, resulting in opening new pathways facilitating drug release more deeply into the skin [51]. Furthermore, the presence of intact elastic vesicles within the SC has been demonstrated, but claims that intact elastic vesicles can permeate through the viable epidermis to the blood circulation are more controversial [52].

Transethosomes have been developed as a combination of deformable liposomes (designed with edge activators also called transferosomes) and ethosomes. The edge activators in transferosomes, such as span 60, span 25, span 80, tween 20, tween 60, tween 80, sodium deoxycholate, and sodium cholate, can be helpful to make vesicles more flexible, but are still unable to deliver drugs more deeply, so synergism along with ethanol can solve this issue [53]. The skin permeation of nano-sized formulations has always remained conflicting. To make ethosomes a realistic approach, the microneedle-driven delivery of different ethosomes has been investigated, since authors have claimed that all nano-carriers are not able to cross SC barriers. Therefore, the utilization of microneedles of appropriate sizes is helpful to cross the SC and release the drug into the probable deepest layers of the dermis [54]. It is very important here to underline that even after crossing the SC, the role of edge activators (Tween^®^80 and sodium cholate) and ethanol cannot be denied, which makes the transethosomes more preferable among other nano transporters. More precisely, cationic nano-carriers with ethanol and edge activators have been chosen as the most promising transdermal drug delivery system [54].

#### 2.1.3. Solid Lipid Nanoparticles (SLN) and Nanostructured Lipid Carriers (NLC)

As described in literature, lipid nanoparticles can be divided in two branches, namely SLN and NLC, in the course of their scientific evolution. SLN with a diameter between 10 and 1000 nm have emerged as an alternative to colloidal or vesicular drug delivery systems comparable to liposomes, lipid emulsions, and polymeric nanoparticles. They contain physiologically tolerated lipids dispersed in an aqueous phase containing stabilizers. Due to their ability to embed both hydrophilic and lipophilic molecules, they became outstanding prototypes for controlled and targeted drug delivery [55]. The biomimetic property of SLN is valuable for penetration through various biological barriers. These characteristics of lipid nanoparticles make them an effective drug delivery system, particularly for lipophilic active moieties that also offer distinguishing properties such as a small diameter, large surface area, and high drug loading [56]. SLN are O/W type nano-dispersions, in which dispersed lipid nanoparticles constitute a heterogeneous system with an inner lipid phase and an outer aqueous phase, stabilized by one or two surfactants [57].

In order to establish synergistic results in the delivery of different active compounds, combination therapy is a much-appreciated approach. This concept of co-delivery is also feasible in the case of lipid-based nanoparticles. Recently, clotrimazole and alphalipolic acid were entrapped into cationic lipid-containing SLN. A prolonged release was recorded without any burst effect. In-vitro testing on 25 strains of *Candida albicans* demonstrated that the anti-microbial capacity was well maintained on loading into SLN. Thus, the findings of studies agree that topical dual drug delivery through SLN as vehicle is an effective approach against microbial infections related to C. albicans [58].

However, a limit of SLN is represented by their physical instability. In fact, during storage, the solid lipids constituting SLN are subjected to crystallization. The formation of a rigid core takes place, limiting the movement of active molecules within the core, resulting in the expulsion of the drug into dispersion media. This serious problem of instability affects the entrapment efficiency of the system [59]. For further understanding, the theoretical models of SLN and NLC have been established using sophisticated analytical techniques including differential scanning calorimetry (DSC) and small-angle X-ray scattering (SAXS). These techniques are capable of identifying the polymorphic forms of lipid matrix, which are dependent on the specific ratio and composition of lipid and surfactant used. Moreover, Cryo-transmission electron microscopy (Cryo-TEM) describes the morphology of nanoscale objects [60]. The polymorphic state of the lipid depends on its compatibility with other oils and stabilizers. Crystallinity throughout the manufacturing process of lipid nanoparticles and their storage determines the drug incorporation and its release pattern [61]. The crystallization pattern of the lipid inside the nanoparticles is different compared to that in bulk. Crystallization is highly dependent on the critical crystallization temperature required to obtain rigid nanoparticles after the homogenization step—liquid state particles (supercooled emulsion) will be produced if a specific temperature is not reached. The choice of surfactant is another crucial factor that influences the kinetics of the polymorphic transition of lipids [62]. The inappropriate concentration of lipid and surfactant can affect the overall internal structure of lipid nanoparticles. This transition from b’-form to b-form could be responsible for drug expulsion. A smart way is adopted in designing the SLN (by controlling temperature and water loss) with the initiation of transformation from a- to b-forms [63]. Thus, it has been drawn as a conclusion that drug incorporation decreases in the following order:

**Supercooled melt < a-form < b’-form < b-form**

The cutaneous utility of lipid nanoparticles holds several benefits, such as the prevention of chemical degradation of the active, the increased deposition in the stratum corneum, and the reduced flux, attributed to some very interesting outputs such as prolonged contact with the skin surface and film-forming properties to prevent water loss. The mechanism of mixing lipid nanoparticles and lipids of stratum corneum promotes the penetration of the active into deeper tissues. Lipid nano-transporters can establish adjacent contact with the superficial junctions of SC and furrows between corneocyte islands, allowing a uniform distribution of the drug. They also offer a widening of inter-corneocyte gaps [64]. Because of physiological lipid composition, SLN/ NLC offer an easy attachment to the SC on forming its lipid rearrangement, allowing embedded drug molecules into the deeper skin layers. Furthermore, their nanoscale diameter also contributes to the enhancement of the influx through the skin. Nonetheless, the adequate selection of lipid composition concerning the molecule’s physicochemical properties also significantly affects skin penetration. The film-formation, namely an occlusive film property of SLN/ NLC on the SC surface, avoids the evaporation of water and also fills the imperfections in the skin [57]. Furthermore, Lademann and co-workers have concluded in one study that the hair follicles (particularly in the scalp, calf, and forehead regions) are better drug depots than the stratum corneum. They have considered that nanoparticles are well suited for driving the drugs into deeper functional structures and even accumulate for some days [65].

#### 2.1.4. Monoolein Aqueous Dispersions (MAD) and Cubosomes

Monoolein aqueous dispersions (MAD) are lipid dispersions able to provide matrices for sustained drug release. Particularly, MAD are heterogeneous systems obtained by dispersing in water an amphiphilic lipid, such as monoolein. MAD are typified by a mixture of complex lyotropic liquid crystalline nanostructures like micellar, lamellar, hexagonal, and cubic phases. It has to be underlined that the prevalence of one nanostructure over another mainly depends on both temperature and system water content [66,67].

Indeed, surfactants and lipids can undergo various assemblies resulting in micelle or lyotropic crystalline phases by hydration. The packing parameters or spontaneous curvature represents the type of assemblies. For instance, monoolein (unsaturated long-chain monoglycerides dispersed in water) has a tendency to create bicontinuous cubic phases on addition of water, resulting in being a good candidate to increase solubility and responsible for controlled and precise drug delivery [68]. A recent study describing MAD to deliver quercetin showed that that quercetin was completely associated with the dispersed phase [69]. Furthermore, the choice of emulsifier with optimum concentration has been studied, where sodium cholate was employed at two different concentrations, 0.15% and 0.25%, with respect to the total weight of the formulation. Certainly, MAD formulated with the lowest percentage of sodium cholate were represented as a mixture of vesicles and cubic structures, whereas MAD with the highest concentration of emulsifier have shown the unilamellar vesicular structures. Interestingly, MAD produced with different concentrations of emulsifier were able to retain quercetin more than 65% after 100 days of the storage period. Quercetin containing MAD can be proposed to attain semisolid products to treat skin disorders such as psoriasis or dermatitis [69]. In a similar manner, a comparative study on the delivery of two flavonoids, namely quercetin and rutin, via MAD has been proposed. It has been confirmed by Cryo-TEM that sodium cholate concentration affects the morphological aspect of MAD. Moreover, diffusion studies have revealed that both MAD systems were suitable for cutaneous applications and the presence of quercetin or rutin did not affect the structural organization of MAD [70]. In another study, it was reported that monoolein/poloxamer/sodium cholate mixtures were suitable for achieving the high encapsulation of curcumin and to avoid the drug degradation for six months. Xanthan gum, selected in order to make the MAD dispersion more viscous, controlled the release of curcumin very efficiently [71].

The emulsification of the cubic lipid phase (transparent and isotropic phase’s crystals) into the aqueous phase makes the system physically stable. They can be administered via various routes such as oral, parenteral, and percutaneous and are biocompatible and can control the release of drugs very efficiently [72]. Cubosomes dispersions have been chosen to entrap indomethacin by emulsifying monoolein and Poloxamer 407 in water. The study of cubosomes’ inner structure conducted by Cryo-TEM revealed the typical ordered cubic texture, while the presence of indomethacin did not influence the ultrastructure of the disperse phase. The prolonged anti-inflammatory activity was shown by cubosomes with indomethacin, and tape stripping has helped in quantifying the decreased amount of indomethacin by time ensured the successful permeation of the system across stratum corneum [72]. Furthermore, the role of two alternate emulsifiers, namely sodium cholate and sodium caseinate in the cubosome production, was studied for the cutaneous application of crocin [73]. The sodium cholate has produced transparent dispersions while the combination of both aforementioned emulsifiers has given an opaque milky appearance. The encapsulation efficiency was more than 80% in both cases. Concerning the internal structure, the transition in morphology took place from the cubosomes to hexosome upon the addition of crocin. No aggregation phenomena have been observed during the six months of the storage period. As per literature, sodium caseinate is known for its own antioxidant activity potential—even empty cubosomes with sodium caseinate have shown some antioxidant activity. Hence, this kind of composition can be considered as a new strategy to vehiculate the crocin and to protect it from degradation [73]. Similarly, cubosomes have been designed in order to deliver curcumin by cubosomes, and the various mixtures of sodium cholate, sodium caseinate, bentonite, and poloxamer have been employed. The resulting dispersions characterized by vesicles, cubosomes, and sponge type phases, based on the composition, were investigated by cryo-TEM and X-ray studies.

#### 2.1.5. Gene Delivery

Gene delivery involves the delivery of nucleic acids such as DNA, RNA, or antisense oligonucleotides to appropriate cells [74]. The gene therapy mechanism is based on the incorporation of a gene that encodes a functioning protein significantly involved in the cure or prevention of any disease progression. Certainly, gene modulation seems to be a very promising method; however, it has some obstacles regarding its delivery to the desired site. The main limitations are rapid clearance, site-specific targeting, and degradation by nucleases, which affect the serum half-life of siRNA to 5–60 min and unmodified DNA to 10 min [75]. However, ligand approach or hydrodynamic injection can minimize these shortcomings, well-designed biocompatible vehicles with the capability to escape from the removal by the immune system, enhanced transgene expression is still required [75]. Moreover, obstructions in non-viral gene delivery are linked to DNA that can be lost due to a lack of strong complexation with cationic lipid, and DNA–Cationic lipid complex can be eliminated from the circulation before binding to the cell surface. Additionally, the internalization of the complete complex is uncertain after invading the cell membrane. Following endocytosis, a fraction of delivering DNA may be degraded because of acidic pH or cytoplasmic DNAse. Improvement in transfection efficiency can be attained with modification in particle chemistry, size control, surface charge, shape, and ligand modification [76]. Advancements in nanocarriers offer plentiful possibilities and the flexibility to choose a target [6]. Due to biodegradability, ease in synthesis and functionalization, and scale-up production, polymers could be an alternative. Moreover, the use of PEGylation to promote polymeric nanoparticle interactions at the cellular level is a well-defined mechanism. However, certain limitations are also associated with the use of polymers that cannot be avoided, including the high costs of manufacturing adding on biological analyses and process development, as well as the lack of understanding of the mechanism of degradation that leads to a generation of toxic metabolites. Furthermore, the toxicity and stability of polymers in a protein-rich biological medium are still conflicting [77]. Notably, some investigations conducted on lipid nanocarriers for gene delivery eliminate all these problems. For instance, a cationic surfactant composing liposomes conjugated with lambda DNA exposed to endo and exo-nucleases present in serum remained intact and stable without causing any toxicity. Thus, transfection by liposomes is a well-established technique [78].

### 2.2. Diagnostic Applications

**Figure 1:**
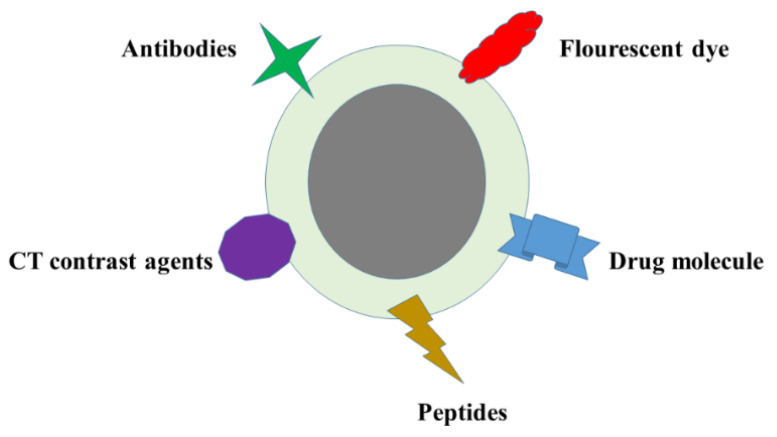
Schematic representation of functionalized MNP. The surface of MNP can be functionalized by the insertion of specific molecules, such as antibodies, peptides, drugs, fluorescent dye, and/or contrast agents for computerized tomography.

Concerning diagnostic applications, nanotechnology has gained considerable interest. The imaging procedures are categorized based on morphological, functional, and molecular levels, which provide the biological detail of a disease with a non-invasive approach. Nano-sized particles are spherical entities made of inert silica, metals, or crystals. In particular, Magnetic Nanoparticles (MNP) are an interesting tool with numerous biomedical applications, including magnetic hyperthermia, cell separations, magnetic resonance imaging (MRI) to track tumor cells or lesions by comparing the overall magnetic response of MNP between pathological and normal tissues, tissue engineering, and drug delivery to very specific areas. MNP can be categorized as pure metals, metal oxides, and magnetic nanocomposites. Moreover, MNP—primarily composed of Co, Fe, Ni, Ti, iron oxide, and some ferrites (BaFe_12_O_19_ and CoFe_2_O_4_)—have gained the highest attention in the biomedical field [79]. In general, magnetic materials are considered as multi-magnetic arrangements within one structure. However, after conversion to the nanoscale with diameters of around 10–15 nm, these magnetic materials act as a single magnetic domain structure. They are responsive only under the influence an external magnetic field, indicating their paramagnetic behavior. Therefore, they can be controlled very precisely via an external magnetic field [80]. The MNP are available in three major forms named as magnetite (Fe_2_O_3_), maghemite (γ-Fe_2_O_3_), and hematite (α-Fe_2_O_3_). Magnetite (Fe2^+^ and Fe3^+^ ions in the 1:2 ratio) is primarily preferred among the three forms of iron oxides. If ferromagnetic material is exposed to an external magnetic field and later turns it off, it still holds the magnetization for a short period, which gives the possibility to control heat and magnetic effect in-vivo [81]. Hyperthermia is a well-established phenomenon, whereby applying high-frequency alternating magnetic field results in heat production by MNP. This transformation of the electromagnetic energy into heat due to MNPs oscillation is very useful in cancer therapy, as tissues exposed to conditions of high temperature (41–47 °C) may undergo apoptosis of the tumor cells. However, the precise control of magnetic heating can be accomplished by controlling some nano-magnetic parameters such as size (V), anisotropy (K), saturation magnetization (MS), and coercivity (HC) during the entire process, but could also damage neighboring cells. The principle of heat diffusion should cause the maximum destruction or killing of tumor cells by avoiding the damage of normal cells [82]. It is worth noting that surface-engineered MNP can be useful for various purposes, including the linking of antibodies, specific peptides, drug molecules, fluorescent dye, and computerized tomography (CT) contrast agents (Figure 1). More than one target can be achieved simultaneously on a single delivery of MNP in the body, as shown in Figure 1 [83].

In addition, the pivotal concept of artificial engineering has been explored based on the iron oxide nanoparticles as an ultra-sensitive nanoprobe, where antibodies can be linked to MNP. The proposed biomarkers could enhance the real-time visualization of certain biological events including cell trafficking, cancer metastasis, and cellular signaling at both the molecular and cellular levels [84]. Apart from various advantages, MNP also experienced certain challenges, including rapid agglomeration due to large surface-to-volume ratio, chemical reactivity, and high surface energy, all of which resulted in magnetism loss. Hence, the improved functionalization of MNP by applying a suitable coating can make them more biocompatible for in-vivo use [85]. For instance, Byoun et al. have modified MNP by incorporating them into the silica matrix and functionalize them with a fluorescent dye. The multi-Fe_3_O_4_@SiO_2_ nanoparticles have outstandingly retained dual functioning, namely fluorescence and magnetism. Moreover, MNP below 10 nm with paramagnetic behavior were susceptible to rapid clearance from the kidney because of a smaller size. Conversely, the increased dimensions with the application of polymer or lipid as a coating material have been proved to be supportive to maintain a longer shelf life in the blood circulation [86]. Some most explored coating material for MNP are dextran and PEG [87], dextran-spermine biopolymer [88], β-cyclodextrin, chitosan [23], and poly (lactide-co-glycolide) (PLGA) as a magnetic core surrounded by a folate-chitosan conjugate shell [89]. Moreover, some techniques, such as electrospinning [90], can also be considered since natural and synthetic nanoparticles may have a significant impact on the production process.

## 3. Parameters to Consider for the Characterization of Nanoparticles

The physical evaluation of nanoparticles is the foundation of their further utilization. A good understanding of physical parameters could be helpful in foreseeing the in vivo behavior of nanoparticles with improved efficacy [89]. For example, the stiffness of nanoparticles can affect bio-distribution patterns, resulting in inadequate internalization and finally drug resistance. Li and Zhang have tailored the hardness of different nanomaterials, namely polymeric nanoparticles, liposomes, dendrimers, and solid nanoparticles, and demonstrated that only rigid nanomaterial can attain better and thorough endocytosis. The possible explanation could be that the shape deformation of soft nanomaterials occurred due to the receptor–ligand interaction. Unlike the case of liposomes, where their penetration into the lipid bio membrane facilitates the interaction between the hydrophobic segments of the liposomes and the lipid tails. Furthermore, the feeble interaction between nanomaterial and drug molecules could give rise to the leakage of drug molecules before their internalization [91]. Soft biological substances cannot be phagocytosed by macrophage and they hardly undergo full wrapping [91,92]. The alterations in stiffness can be evaluated by changing the cross-linking density in order to modify the hardness of hydrogel particles. In the case of liposomes, stiffness considered by particle stability depends upon the diameter, phospholipid composition, and presence of cholesterol. The hardness of liposomes is inversely proportional to size while directly proportional to the transition temperature of the liposomal bilayer [68]. Nankano and Tozuka have reported a unique method to assess the hardness of liposomes by combining atomic force microscopy (AFM) and dynamic light scattering (DLS) techniques [68]. Therefore, hardness and softness are both crucial parameters that diverge based on the target and type of delivery system.

**Figure 2:**
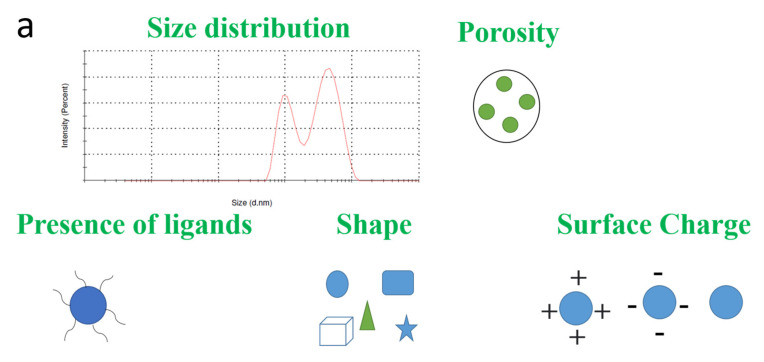

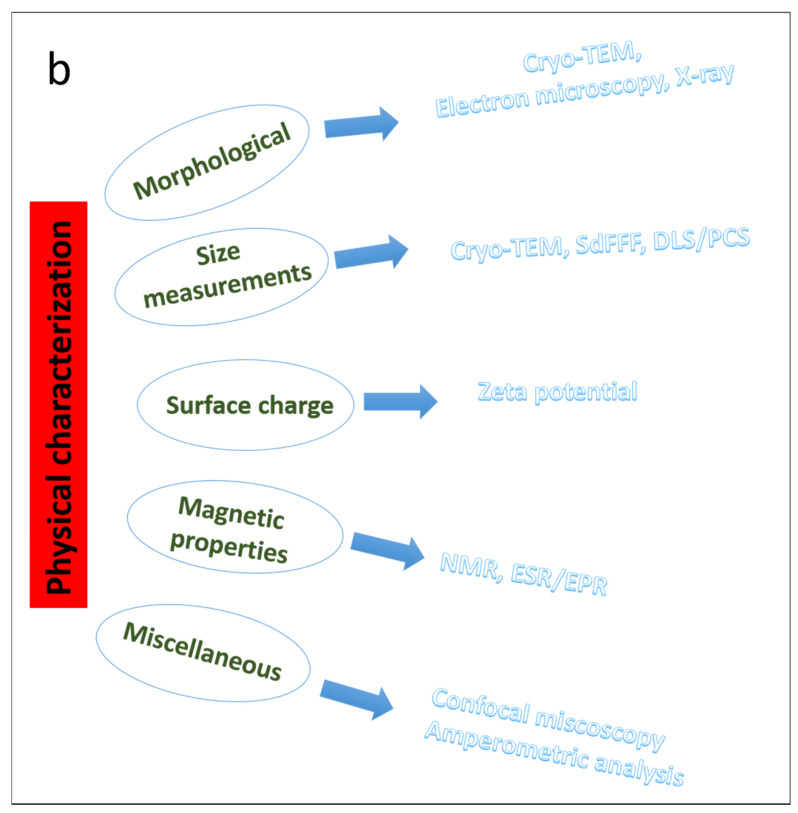
Schematic resume of the principal parameters (**a**) and techniques (**b**) used for nanoparticles characterization.

Other physical aspects such as size, shape of nanoparticles, agglomeration state, and growth kinetics can be confirmed by TEM; structural properties, crystal structure, and element chemical composition can be confirmed by XRD. Furthermore, the charge on the surface can be measured by zeta potential measurements, whereas the 3D visualization of nanoparticles can be possible with SEM and the number of heterosized population of nanoparticles can be traced by DLS [92,93,94]. The various techniques for physical evaluation of nanoparticles are indicated in Figure 2.

The specificity has been always conflicting because more than one technique can be employed to assess a single characterization parameter. Therefore, the shortcomings with scaling up of nanoparticles with their analysis, such as the absence of reference materials for calibration and difficulty in data interpretation, cannot be overlooked [92]. Plenty of analyzing techniques have been approved, but a robust strategy for a complete characterization profile of nanoparticles is still an unmet task. Not only limitations of existing techniques are responsible but also nanomaterial properties lead to the misrepresentation of results. The reason for variability in diameter is based on type of nanoparticles, surface area, and surface charge density [95]. In depth, surface properties highly depend on a nanoparticle’s microenvironment, functional groups, and reactivity; finally, aging can also be responsible for occurrence of several abnormalities such as swelling, shrinking, depletion of impurities on surface, agglomeration/aggregation, and multipolar deformations.

The diameter of the nanoparticles can be defined as the inversely proportional relationship between surface, volume, and surface energy of the nanomaterial, which has a higher energy state than the bulk material. Indeed, it has been recognized that most of the atoms in the nanomaterial remain near or on their surface, so the surface composition and structure of the nanoparticles play a crucial role during storage and exposure to different environments [96,97,98].

More importantly, the size is not associated only with the core of nanoparticles but also with the substances adsorbed on the surface of nanoparticles such as stabilizers and the mobility of electrical double layer (solvation shell) along with the particle. The thickness of the electrical double layer and its impact on the final results depends on the substances present in the colloid and on the surface of the nanoparticles [93].

The fate of lipid nanomaterials is a very important aspect highly relevant to physical change over time. In this manner, physical evaluation should be performed at every step of nanoparticle development (from the preparation to storage of nanoparticles) to understand the degradation pattern of nanoparticles.

In Figure 2, various physical characterization techniques for nanoparticles have been given. The main parameters to be considered are size distribution, porosity, surface charge, aggregation, dispersion ability, etc. The porosity has great relation with zeta potential values. In the case of mesoporous silica nanoparticles, alteration on pore size and porosity can result in the substantial reduction in zeta potential up to 25% lower than the theoretical zeta potential predictions for a flat surface at the corresponding ionic conditions in moderate pH range. The zeta potential values for pore openings are different from the solid surface around mesoporous silica nanoparticles [99].

Furthermore, the comparison and utility of techniques have been discussed in order to make a protocol for better evaluation of nanomaterials [93]. The morphology of nanoparticles can be investigated by TEM, SEM, and X-ray studies while the size can be measured using DLS, SdFFF, and optical microscopy. To understand surface charge chemistry, zeta potential measurements are helpful. However, NMR, ESR, and confocal microscopy can be useful to establish a complete understanding of physical parameters as well as in vivo investigation of nanoparticles.

## 4. Morphological Characterization of Nanoparticles

### 4.1. Cryo-Transmission Electron Microscopy

Electron microscopy allows the direct visualization of nanomaterials; therefore, it is an important means of investigating nanosized pharmaceutical forms. This technique allows the obtaining of information about the shape, inner structure, and surface of particles that could not be identified otherwise.

**Figure 3:**
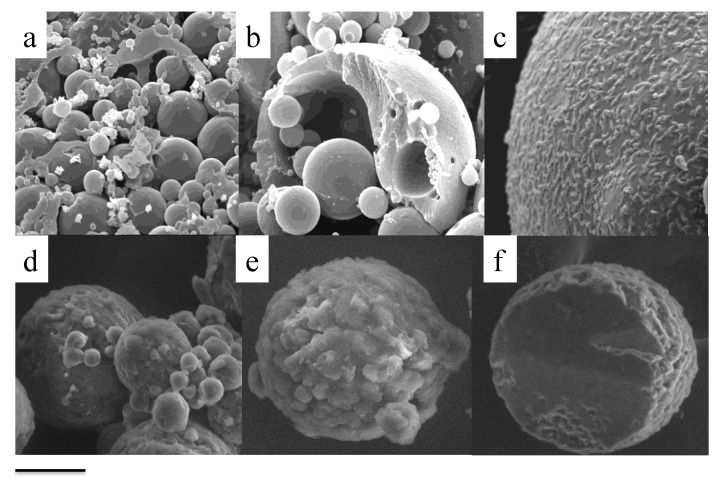
SEM photographs of polymeric microparticles, namely eudragit RS (**a**,**b**) and hydroxypropylmethylcellulose (**c**), and lipid-based microparticles, namely tripalmitin:glyceryl monostarate 2:1 by weight (**d**–**f**). Bar represents 20 μm in panels (**a**,**b**), 10 μm in panel (**c**,**d**), and 40 μm in panel (**e**,**f**).

Particularly, scanning electron microscopy (SEM) is useful to investigate the shape, surface, and size distribution of dry microparticles [100], and, more importantly, it can allow the visualization of the outer and inner structure of sectioned particles, therefore obtaining information about the organization of the components of the particle and verifying if the particle is a “sphere” or a “capsule” (see Figure 3).

**Figure 4:**
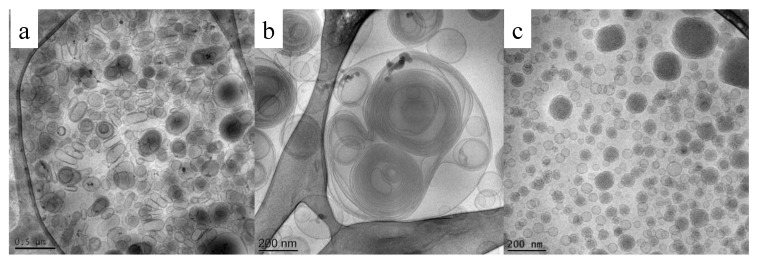
Cryo-TEM images of empty liposomes (**a**), ethosomes (**b**), and cubosomes (**c**).

Cryogenic transmission electron microscopy (Cryo-TEM) is a useful method for analyzing liquid dispersions in a very close way to their native state, revealing morphology and inner details. Milky nanosized liquid dispersions can be accurately identified by cryo-TEM. Indeed, the possibility of vitrifying the samples brings the frozen sample very close to what the real dispersion is [101]. Uni- or oligo-lamellar vesicles, lamellar phases, cubic phases, cubosomes, exosomes, etc. can be visualized and measured one by one (see Figure 4).

In the case of vesicles, especially ethosomes, multilayered vesicles have been observed because of lamellar organization exhibited by phosphatidylcholine. Furthermore, unilamellar or multilamellar structure depends upon lipid/surfactant composition of phosphatidylcholine bilayer system [32,102].

A very short wavelength of accelerated electrons can provide very high resolution imaging [103]. The morphology of nanosystems is highly dependent on the type of lipid and other components employed. The study aimed at production of NLC using different solid lipids (i.e., tristearin, compritol, precirol, or suppocire) and a liquid lipid (i.e., caprylic/capric triglycerides) for the cutaneous delivery of α-tocopherol. The NLC morphology was investigated via cryo-TEM and the shape appears discoid in the top view and more electron-dense and rodlike in the edge-on view, while a roundish shape has been observed in the case of NLC made up of tristearin, both for unloaded and loaded NLC. Ovoid and triangular structures were observed in NLC with compritol and precirol. Lastly, in the case of NLC with suppocire, besides the presence of some irregular structures, spherical structures were detected, resembling vesicles rather than solid particles [94].

**Figure 5:**
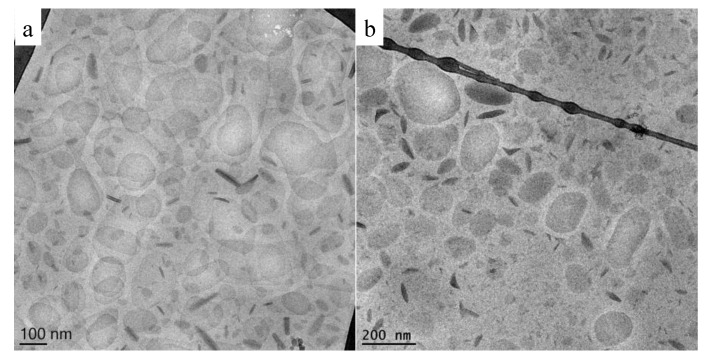
Cryo-TEM images of SLN (**a**) and NLC (**b**).

Figure 5 shows cryo-TEM images of tristearin SLN and tristearin-Miglyol812 NLC dispersions [104]. The particles are projected in two-dimensions; therefore, on the basis of the angle of observation in the vitrified samples, the particles appear differently assuming hexagonal, elongated circular platelet-like (top view) or “needle”-like forms (side views). It should be underlined that the NLC side view (needles) seem more elliptically shaped compared to the SLN side views, likely due to the presence of the oil component (Miglyol 812) forming compartments sticking to the surfaces of the nanoparticles solid matrix [105,106]. The nanoparticle thickness is generally between ≈5 and 40 nm, but it is very difficult to measure it exactly due to the tilt of the particles.

**Figure 6:**
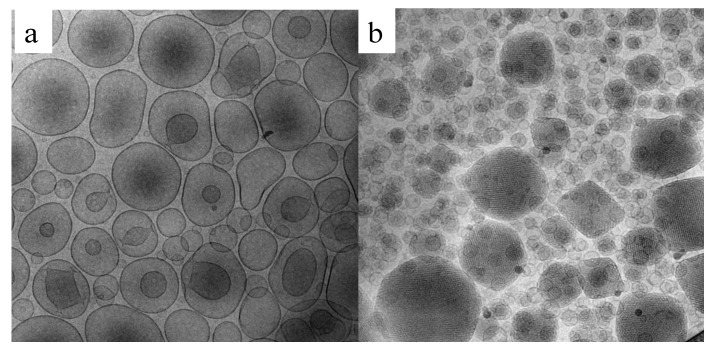
Cryo-TEM images of MAD obtained with hydrotrope (**a**) or hot homogenization (**b**) method in the presence of poloxamer 407 [73]. (With Elsevier permission, License Number 5785341232886).

Cryo-TEM images were also useful to discriminate the morphology of MAD obtained following two different methods of preparation, namely hydrotrope or hot homogenization. The hydrotrope method consists of the use of compounds, such as sodium cholate, that are able to solubilize hydrophobic molecules in water by means other than the formation of micelles. As shown in Figure 6, it is evident that—notwithstanding what is reported in literature by some researchers indicating that both methods resulted in the production of cubosomes when poloxamer 407 is used as surfactant—in the case of the hydrotrope method, uni- or bi-lamellar vesicles were obtained [73,107,108].

On the other hand, the dispersion obtained by the hot homogenization method gave rise to the formation of coexisting vesicles, spherical, and cubosome particles. The presence of vesicles fused on the surface of cubosomes possibly undergoes well-ordered particles over time [72,109].

The images taken from cryo-TEM observation are useful also to measure particle dimensions by a scale bar. Particularly, it is evident that two different populations are often present: the former is mainly of large cubosomes and the latter of smaller cubosomes and vesicles of 100 nm and below.

Indeed, the most important benefit of microscopic technique is that it is feasible to know about the morphology and the diameter of the particles within the same run. However, preparing the sample is very important—nanoparticles should cover the substrate precisely and reagents used in sample preparation should be appropriate [110,111]. In addition, it should be mentioned that microscopic techniques used for the characterization of nano-sized materials could be challenging if the sample to be analyzed is polydispersed due to aggregation.

### 4.2. X-rays Diffraction Studies

X-ray diffraction studies were frequently conducted on lipid dispersions in past decades [112,113]. It is well known that the morphology of nano-sized drug carriers directly influences the release and encapsulation efficiency of the active molecule. Therefore, an investigation of the internal structural organization of nanosystem becomes very important. X-ray diffraction analysis is the most explored tool to achieve this task. Generally, in lipid-based formulations, lipids can aggregate into many structures via interplay parameters including temperature, water concentration, or the presence of different components. The phase stability is described by these lipid arrangements. Lipid phases are most often detected in drug delivery nanosystems such as lamellar, hexagonal, and cubic phases [114].

The number of phases observed in lipid-water systems as a function of composition and temperature is quite large [67,115]. The phases can be described and classified according to different criteria. In this case, a reference to the ability of lipids to segregate in water into specific regions (called “structure elements”) is considered together with the position in which they combine a periodically ordered long-range organization of the structure elements (in 1, 2, or 3 dimensions) and a highly disordered short-range conformation of the hydrocarbon chains (see Table 2).

The structural elements of the lamellar phase are lamellae and the phase is described as an ordered 1D succession of planar sheets of lipid and water. The hexagonal phase is characterized by cylindrical micelles packed in a 2D hexagonal lattice. Particularly, in the type I phase (direct), the hydrocarbon chains are inside the cylinders and water is outside, while in the type II phase (inverse), each cylinder contains water and the region between the cylinders is filled with the hydrocarbon chains. Cubic phases are a mixture of micellar and bicontinuous cubic phases [116]. In cubic phase type I, micelles are filled with hydrocarbon chains and separated by water (Pm3n); while cubic phase type II is characterized by water inside and hydrocarbon chains outside (Fd3m). Bicontinuous cubic phases are described as convoluted (folded) surfaces and presented as the infinite periodic minimal surfaces (IPMS) [67,115]. However, their structure is conveniently visualized as two 3D networks of connected rods, mutually intertwined and unconnected [116]. Depending on the type of rod junction, different IPMS are obtained, namely gyroid G-surface for co-planar junction 3 by 3 (Ia3d cubic phase), diamond D-surface for tetrahedral junction 4 by 4 (Pn3m cubic phase), and primitive P-surface when the rods are cubically joined 6 by 6 (Im3m cubic phase). The Ia3d bicontinuous cubic phase exists either as type I (oil-in-water) or type II (water-in-oil).

It is very important to verify the impact of presence of drug molecules on the overall structural organization of nanosystems. The scaling up study aimed at fabricating progesterone-containing NLC and SLN at a pilot scale has been accomplished, where the lamellar structural organization of lipid nanoparticles has been confirmed. Furthermore, the obtained data indicated that the presence of progesterone has slightly affected the unit cell dimensions [117]. In another study, two different compositions have been employed for the production of NLC in order to embed ellagic acid for dermal delivery. Tristearin was solid lipid in both cases while liquid lipid tricaprylin and labrasol have been chosen alternatively. The influence of different lipid blends on overall stability, release profile, and activity have been examined. In SAXS studies, loaded as well as unloaded NLC preparations were very similar; therefore, it can be emphasized that neither the different lipid composition nor the presence of ellagic acid as a drug molecule alter the structural organization of lipid nanoparticles. Note that considering the very low solubility of ellagic acid in water, the data suggest a solubilization of the active inside the paraffinic region of the lipid layer [118]. Furthermore, the structural organization of both SLN and ethosomes has been studied for the delivery of caffeic acid. In the case of unloaded and loaded SLN, a bilayer-to-bilayer repeat distance has been calculated from Bragg peaks, independently from the presence or the absence of caffeic acid. Conversely, in the case of ethosomes, very disordered positional correlations between adjacent bilayers and/or few stacked multi-lamellar structures have been traced out. Interestingly, the addition of caffeic acid modifies the surface charge density of the ethosomal bilayers and balances the attractive and repulsive forces between adjacent membranes. As found by other authors, the association of phenolic compounds with vesicular systems organized in bilayers can positively influence the bilayer organization [32,119]. The Cryo-TEM results can corroborate the SAXS results, giving information on the morphology and structural organization of the nanomaterials. Furthermore, our recent study aimed to design liposomes for targeting synthetic quorum sensing inhibitors, using different surfactants (either anionic and cationic) to reach the best composition and activity against P. aeruginosa biofilm formation showed that by small-angle X-ray scattering, it was possible to see the effect of type of charged surfactant on the structural organization of liposomes by getting different types and number of bands. For example, in the case of plain liposomes, a multi lamellar structure was found, while in the case of cationic surfactant, there was a loss of positional correlations between adjacent bilayers, likely due to surface charge density conferred to the bilayers upon surfactant insertion. Hence, unilamellar structures have finally been observed in the case of anionic surfactants, in which the absence of a scattering signal observed indicates a complete loss of structure [102].

In this way, the fusion between the TEM and X-ray results can provide information on the internal structure but also on the localization of lipids and drugs within the nanostructure.

## 5. Size Measurements

Commonly, the size of nanomaterials being utilized for drug delivery purposes ranges from 10 to 200 nm. These size specifications can be considered as per molecular filtration size and the reticuloendothelial system uptake cut-off [120]. Moreover, it is very important to determine the strengths and weaknesses of employed methods for nanomaterial characterization in order to distinguish their limits; for example, analytical ultracentrifugation and flow field fractionation methods are disadvantaged by objects like too minuscule particle size owing to the particle charge [121]. Diameters can be measured by various techniques such as Sedimentation Field Flow Fractionation (SdFFF), Photon correlation spectroscopy (PCS), and (once again) cryo-TEM.

### 5.1. SdFFF

**Figure 7:**
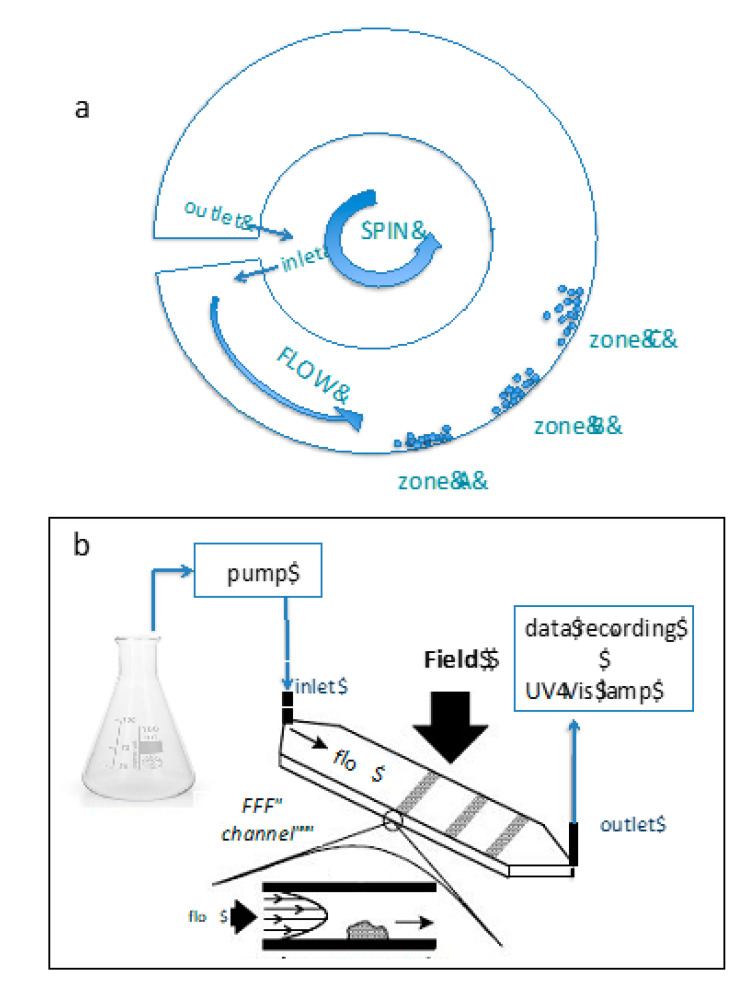
SdFFF operation diagram (**a**) and instrument configuration set up (**b**).

The Sedimentation Field Flow Fractionation (SdFFF) technique is meant for the separation and characterization of nanoparticles based on gravitational or centrifugal force as an external force (see Figure 7).

Particularly, in FFF techniques, the separation occurs by the retention of the particles inside the channel, retention that is due to the application of an external field perpendicular to the flow and counterbalanced by the diffusion phenomenon in the liquid carrier. The factors most influencing the FFF analysis can be summarized as (a) sample preparation, (b) properties of the liquid carrier, (c) channel size, and (d) influence of the applied external field [122].

SdFFF is not only limited to analyze size distribution but also useful in various tasks such as the fractionation of proteins (40–300 nm), nucleic acids (<70 nm), polysaccharides assemblies (0.1–1 µm), cells (10–20 µm), and virus-like particles (10–80 nm) [123,124].

In one study concerning lipid-based fluorescent nanoparticles designed to access in-vivo biodistribution via fluorescent luminescent imaging and in vitro uptake on hCMEC/D3 cell line, SdFFF was utilized to determine the size distribution of particles by converting the fractograms and it was useful to study the influence of different compositions on the overall diameter. For instance, authors were able to confirm that the lipid component and the presence of both fluorescent and tween 80 slightly affected nanoparticle dimensions [110]. Furthermore, the attained fractograms at fixed density values have been converted into PSD plots (the amount of material per unit change of diameter, according to well-verified equations) by transforming the retention time in a size.

Similarly, NLC with different protocols have been prepared to embed a prototypical cannabinoid drug [111]. In order to get more accurate results, the data obtained from PCS have been corroborated with SdFFF. Both the data agreed with each other very well in terms of the number of populations detected by both techniques. Changes in the composition and passage of production of NLC (by alternatively adding the lipid phase into the aqueous (direct protocol) or the aqueous phase into the lipid (reverse protocol) have shown very similar patterns with both the techniques [111].

### 5.2. Dynamic Light Scattering/ Photon Correlation Spectrometry

The measure of diameter is a crucial step to distinguish nanoparticles from bulk materials. Particularly in DLS, the laser light scatters when it passes through a colloid. The intensity of the inflected scattered light is determined as a function of time, and the hydrodynamic diameters of particles can be measured [125].

Charge on the surface of nanomaterial and their dimensions are the two most important parameters to be considered, as they are highly responsible for several biological factors including cellular uptake, toxicity, and dissolution. These factors are subsequently accountable in drug release at the target site. Furthermore, biological matrices could modify both parameters with different mechanisms, such as protein adsorption causing the characteristic corona or the interaction of nanosized particles with certain biological molecules, leading to coronas that manage a certain control and interfere with the living system [126,127].

The mathematical equation derived from DLS results are highly based on viscosity of the solvent, instrument, temperature, and the refractive index of materials [128,129].

Generally, in DLS, autocorrelation function (ACF) of the scattered light can be fitted by the cumulant and/or CONTIN method as a mathematical algorithm and analysis. The cumulant method is where the cumulant term is provided with mean diameter and polydispersity [130]. However, this method is not suitable in the case of heterogeneous polydisperse samples [131]. The difficulty in polydisperse and heterogeneous samples can be overcome using the CONTIN algorithm method. Here, the correlation function is fitted against a longer time duration and gives size distribution analysis with average size and width for every peak, whereas in the cumulant method, the initial part of the ACF is fitted into a single exponential decay [132]. Interestingly, both methods should be suitable for completely monodisperse samples. Therefore, results obtained with these two algorithms are usually different. The polydispersity values obtained from the results describe the intensity of light being scattered by particles of different diameters and is calculated by the ratio (width/mean)^2^ for each peak [131]. Furthermore, there are various parameters that need to be considered that subsequently affect the results; such factors are discussed in Table 3.

Moreover, the detection limits of two sophisticated techniques, DLS and UV-Vis spectroscopy, have been checked on silver nanoparticles and colloids as mixture of nanoparticles with different sizes: 10 nm and 55 nm and 10 nm and 80 nm, respectively. Practically, the potential to observe small objects (10 nm) in the presence of large ones (55 nm and 80 nm) have been focused.

As expected, it was very difficult to characterize polydisperse colloidal particles since the scattered light through bigger particles was more intense than the light coming through smaller ones. Therefore, it was not possible to detect a higher fraction of smaller nanoparticles because of the interference created by a small fraction of bigger particles, limiting the precision and accuracy of the results of both the techniques. Particularly, UV-Vis was not able to separate out the peaks of different diameters of particles. Hence, UV-Vis should not be considered in analyzing polydisperse colloidal systems. The better alternative could be AFM or TEM [99].

### 5.3. Cryo-TEM

The above reported microscopic technique allows at the mean time the achievement of the morphology and the diameter of the particles. In this view, the sample preparation is very important [99,137,138].

It is worth underlining that in the previous work done by our research group, we used all three aforementioned techniques to control the diameter of lipid nanoparticles and we concluded that all the techniques have their own limitations. For example, the diameter of nanoparticles was recorded in the case of SdFFF and largest in the case of PCS and the diameter assessed by TEM was in the middle of both. The possible explanation could be that different principles are involved in different techniques. The comparison and limitations of the techniques are described in Table 4.

## 6. Zeta Potential and Surface Charge

The interface of particles becomes electrically charged when immersed in specific fluid. It possibly takes place because of a sum of mechanisms, such as the adsorption of the charged surfactants to the particle surface or ionization of surface groups that consequently leads to surface charge density. There is no way to measure surface charge directly, but it can be made possible by creating an electrical field around a particle. Surface charge generally measured in terms of voltage [95]. The zeta potential measurement technique is based on electrophoresis. More precisely, particles in their respective suspending media are exposed to the electrical field. Charged moieties will exhibit drift under the influence of this external electrical field (positive bodies will travel toward negative electrodes and vice-versa). Interestingly, the thin layer of ion and solvent around the particle will drift along with the particles [141].

However, getting high-quality results from zeta potential measurements is very challenging and highly dependent upon various crucial factors including the formation of aggregates low signal-to-noise and the blackening of the electrodes can occur at high ionic strength. Upon aggregation, particles become very large in diameter and undergo settlement; subsequently, the suspending media are too diluted to attain adequate signal-to-noise ratio and could not remain dispersed longer. Moreover, the aggregation process is dynamic; thus, it keeps changing with the passage of time. The rate of aggregation can be controlled by modifying the concentration of solution in which the nanoparticles are dispersed [142]. An investigation has been reported, where the reproducibility of zeta potential results has been tested on four different types of nanoparticles. Drastic changes in zeta potential values occurred at extreme dilutions. This shift was not because of change taking place in properties of nano-suspension, but due to the limitation of the instrument because of signal contribution arising from the extraneous particles present in the liquid media.

Hence, basic characterization should be done for every new sample in order to find the best concentration of the sample with reliable reproducibility [143]. It can be pointed out here that the obtained zeta potential results will not change by optimizing the value for concentration at which particle is aggregating. Therefore, zeta values will not fluctuate during analysis and more reliable results can be obtained [142,143,144,145,146,147]. Furthermore, the charge density is associated with particle properties while ionic strength and composition are linked to the suspending medium.

Furthermore, functionalized nanoparticles possess certain functional groups/surface cites that can turn charged moieties on dispersal into water. This charge variation highly depends on the pH of the solution. Indeed, the same group can have a different charge (positively or negatively) at different pH values. For instance, the hydroxyl group turns into protonated/positively charged at low pH and negatively charged at higher pH. These surface sites become neutral at a specific pH called isoelectric pH. Measuring this isoelectric pH is always challenging [148,149]. Furthermore, the properties of active molecules entrapped in nano-carriers can affect the overall pH of the dispersion and finally the zeta potential values. The two carriers, namely SLN and ethosomes, have been designed to load caffeic acid. The pH value of unloaded SLN and ethosomes were around 5.5 and the zeta potential values were −13.83 ± 0.02 and −16.21 ± 4.5 mV, respectively. Interestingly, upon the addition of caffeic acid, the pH values shifted to 3.7 and the zeta potential values were nearby to neutral charge (Table 5). In this manner, negatively-charged surface possessing nanosystems are capable of embedding positively-charged drug molecules. This factor can enhance the overall encapsulation efficiency of drug delivery systems [32,119]. Some examples from literature related to lipid-based nano-sized drug carriers are summarized in Table 5.

## 7. Characterization of Magnetic Nanoparticles

### 7.1. Nuclear Magnetic Resonance (NMR) Spectroscopy

NMR is a very advantageous analytical technique for quantitative and structural architecture analysis of nano-sized materials. The NMR phenomenon is shown by nuclei having non-zero spin under the influence of an external strong magnetic field, which create variation in energy between the spin up and down states. Transition between these two phases can be sensed by electromagnetic radiations. In the field of physical characterization of nanosystems, NMR can be applied in order to investigate the interactions or coordination between the ligand and the surface of diamagnetic or antiferromagnetic nanoparticles [150]. The high-resolution technique becomes a useful tool for gathering information regarding mobile or dissolved constituents, especially in solid-state.

NMR is perfectly suitable to characterize the solid particles in terms of molecular mobility as well as the rotational diffusion of the particles [151]. It can be employed for structural characterization of lipid nanoparticles. Particularly, interaction of entrapped drug molecules with the lipid core can be examined with the help of this tool. With this regard, coenzyme Q10 has been incorporated into a solid lipid matrix of nanoparticles. The association of Q10 with lipids was confirmed by observing spin diffusion pattern between protons of lipid and protons of Q10, which has further assured that more than 60% of drug was found to be associated with lipid matrix while the rest of amount formed separate domains [152].

Furthermore, NMR can be employed to characterize functionalized lipid nanoparticles such as stabilization of nanoparticles via PEGylation. The blend of lipid namely tripalmitin, lecithin, and poly(ethylene glycol) (PEG)-stearate has been used to produce lipid nanoparticles by an emulsification-solvent evaporation technique. Wherein, it was feasible to trace out tripalmitin in the core of nanoparticles as a major component and whereas PEG-stearate was inflexibly adhered to the surface of the nanoparticles, producing a hydrated polymeric layer. On the other hand, it can also be revealed that the density of PEG coating can be altered by the fraction and the molecular weight of the PEG-stearate used in the nanoparticulate dispersion [153].

Additionally, the NMR based approach can be useful in the characterization of gold nanoparticles functionalized with PEG designed for anti-cancer therapy. NMR was basically employed to understand specific intermolecular interactions and the location of the active molecule in the nanoparticles. The analysis revealed the co-existence of two microenvironments of entrapped drugs, namely proteasome inhibitor bortezomib, precisely by binding the PEG portion of the coating and by absorption on the nanoparticles surface. This study could be useful to analyze the sustainability of drug molecules [154]. Furthermore, Nafion-based lipid nanoparticles have been proposed for creating an ionic connection between the perfluorosulfonic acid resin and the protonable phosphine 1,3,5-triaza-7-phosphaadamantane (PTA), suitable for the coordination of platinum. Nafion is a charged surfactant in which the hydrophilic head is typified by the sulfonic functional groups and the hydrophobic portion by the tetrafluoroethylene structure. Therefore, in the lipid domain, the lipophilic portion of Nafion interacts with the lipid core of the particles, while its polar moiety is present on the surface of nanoparticles. The presence of the sulfonic groups on the surface could therefore be used to interact with Pt-coordinated PTA. In this study, the capability of Nafion-containing nanoparticles to selectively protonate PTA on nitrogen and the coordination ability of the NAF/PTAH+ system to Pt was assessed by NMR. Notably, this study could be foundation for loading and detaching of platinum via alterations in pH or ionic strength [155].

### 7.2. Electron Spin Resonance (ESR)/Electrone Paramagnetic Resoanance (EPR)

The use of magnetic moments of unpaired electrons can describe the electrical, optical, and magnetic behavior of the respective materials. This technique is based on resonance absorption of the magnetic components of external electromagnetic radiations by a spin system, useful for characterization of materials having unpaired electrons in the systems. Therefore, samples containing paramagnetic material are well accepted because they can propagate ESR signals [156,157]. ESR can be used to assess magnetic nanoparticles for in vivo biodistribution in the case of drug targeting. This technique is very sensitive and able to detect both endogenous (iron-protein complex) as well as exogenous source of iron. It can quantify magnetic nanoparticles concentration in biological samples. One approach has been explored to quantify the magnetically driven nanoparticles in brain tumor targeted in rats at lowest concentrations approximately 30 nmol Fe/g tissue [158].

One important concern associated with nanomaterial is biological toxicity under certain experimental conditions. The possible reason could be nanoparticles can facilitate electron transfer and produce free radicals. For instance, nanoparticles under biological environment can give rise to reactive oxygen species such as superoxide, hydroxyl radical, singlet oxygen and hydrogen peroxide that are powerful oxidants able to induce damage at cellular level. Hence, ESR can be a promising technique to identify and quantify the ROS generated under chemical as well as biological environment [159].

ESR is not only limited to quantify ROS but can also be employed in order to assess antioxidant capacity of nanomaterials. The hydroxyl ion scavenging by nanomaterials have been assessed via spin trapping technique in ESR. The capacity of nanoparticles to interact with ROS is highly dependent on size because particles with diameter around 9 nm were able to show maximum antioxidant activity. More specifically, antioxidant properties of the gold nanoparticles in oligochitosan solutions depend on the particle size [160]. Furthermore, specific coating of surfactant over magnetic nanoparticles can affect resonance properties. The magnetic nanoparticles have been coated with gold, sodium-oleate and methoxypoly(ethylene glycol), and in all cases the coated magnetic nanoparticles have shown super-paramagnetic behavior with strong surface effect on magnetic behavior of nanoparticles. Moreover, along with particle size reduction, surface to volume ratio increases. This effect was higher in the case of gold-coated magnetic nanoparticles because of the strong interactions between gold atoms and Fe_3_O_4_ particles.

Two important parameters have been also assessed, namely the resonance field (decreasing by coating) and the line width (increasing) of the ESR spectra [161]. Interestingly, ESR can be applied in order to investigate cell penetration and endocytosis performed by nanomaterials. Wherein, the detail about the number of nanoparticles entering the cells and concentration of drug molecules being released within the cell microenvironment can be evaluated. Additionally, as described earlier, it can also be used in order to assess the radical scavenging activity during inflammation or cancer state. It is an interesting point to note down that structure and intensity of ESR spectra keep changing along with the process steps of endocytosis. The various stages could be described as follows. Firstly, when the spin label is not bound to the cell it gives single narrow spectra. Secondly, when the spin label attaches to the surface of the cell a wide triplet is formed. Thirdly, when the spin label is inside the cell (endosome or lysosome), gives a narrow triplet spectrum and fourthly, when the spin label enters inside the mitochondria of the cell the concentration of spin label starts declining because of their reactions with free radicals. The ESR can be useful in various types of cells such as cancer, endothelium and yeast cells. This strategy can also describe the toxicity associated with the nanomaterials [162].

The EPR experiments have been performed to check influence of dibucaine (a long-acting local anesthetic) on organization of lipid core of structural on SLN and NLC by incorporating stearic acid-derivatives spin labels probes into lipid core. Using label probes, it has been suggested that bilayer lipid core has a tendency to retain maximum dibucaine. EPR can also be used to evaluate stability of drug molecules inserted into lipid cores on heating and cooling of nanoparticles. Hence, no lipid reorganization as a function of temperature occurs [163].

## 8. Miscellaneous

### 8.1. Confocal Microscopy

Confocal laser scanning microscopy is an imaging technique used to generate images from cells or tissues, providing higher resolution and at selective depth more beneficial than conventional microscopy or fluorescence microscopy. This technique is based on the principle of striking incident beams with photons at a given wavelength over a sample. The interaction between illuminated photons and atoms from the sample produces new photons of lower wavelength finally identified by the detector. Point by point scanning is possible by reconstructing images in optical sectioning [164]. Thanks to this optical sectioning, it is possible to track the nanoparticles under the skin. Confocal microscopy can enhance the localization, visualization and penetration of nanomaterials into skin [165]. This technique is very much useful in uptake studies. The uptake of cubosomes into fibroblasts and macrophage cell lines (native state with no fixing) has been explored via live cell imaging associated with the confocal microscopy and individual fusion events have been examined through visualization. Furthermore, the results obtained were corroborated very well along with results from fluorescent-activated cell sorting technique. The uptake mechanism of cubosomes has been revealed by confocal imaging in the case of fibroblast via blending of cubosomes lipid bilayer with cell membrane. The fluctuation in fluorescent pattern by multiple time represents cubosomes uptake followed by immediate recycling (transfer to lysosomes) [166]. Similarly, confocal microscopy has been linked to flow cytometric analysis in the case of uptake studies for camptothecin loaded nanoparticles [167]. Since the cancer cells possess numerous portions, they can be considered as excellent targets for therapy and imaging. The main obstacles to drug delivery into these sites are poor blood flow, high interstitial fluid pressure and damaged blood vessels in these areas. These were overcome by designing c(RGDfK) labeled chitosan capped gold nanoparticles and they have shown their enhanced and selective uptake into MCF-7 and HUVEC cells compared with non-targeted chitosan capped gold nanoparticles [168]. Along microscopic techniques, it can be mentioned the possibility of using other for uptake study of ethosomes loaded with Coenzyme Q10 on fibroblast, such as TEM. With this electron microscopy technique, the passage of ethosomes undergoing endocytosis was clearly visible. The results led to conclude that both loaded and unloaded ethosomes did not affect the organization of cell membrane. Therefore, ethosomes are very promising for entering through narrow constriction because of high flexibility [50].

### 8.2. Amperometric Approach

**Figure 8:**
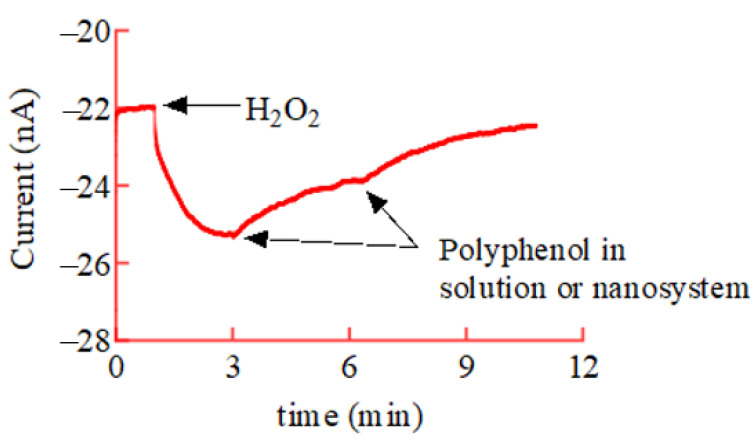
Relative amperometric current response of SCOE dipped in citrate buffer saline, pH 5.5.

To assess the permeation of polyphenols through the skin, an amperometric approach can be used. More specifically, an oxygen electrode coated with pig skin can be used for this purpose, in this case the skin will act as a source of catalase and peroxidase enzyme. This tool can be exploited in order to analyze the kinetics of polyphenol permeation across the stratum corneum and its participation in antioxidant reactions mechanism within the skin in the presence of hydrogen peroxide, H_2_O_2_. More precisely in the skin covered oxygen electrode (SCOE), an electrode allows the registration of variation in oxygen concentration because of hydrogen peroxide-polyphenol reactions in the skin membrane [169,170,171]. After achieving baseline current correspondent to 0.26 mM of dissolved O_2_ in the solution, H_2_O_2_ is added to the buffer system to mimic the inflammatory conditions, wherein the SCOE is already dipped. H_2_O_2_ passes through skin and interacts with catalase enzyme naturally present in skin, giving rise to oxygen production. The oxygen produced from the reaction will be sensed by oxygen electrode and the reduction current will be increased. While in second phase with addition of polyphenol after completion of hydrogen peroxide response will bring decrease in reduction current. The possible reason could be that in the presence of two molecules of H_2_O_2_ and enzyme like peroxidase, polyphenol will be getting oxidized. The sequence of the reaction described up to now leads to the amperometric response reported in Figure 8. This kind of model can be employed to assess antioxidant activity of polyphenols especially when they are entrapped within different nano-sized carriers. This strategy has been employed first time for ethosomes and SLN [119].

Recently, caffeic acid loaded into ethosomes and SLN have been investigated. The release of caffeic acid from respective nanosystems and its further involvement in antioxidant activity has been studied [119]. Interestingly, ethosomes were more promising over SLN and have shown fast release of caffeic acid. Additionally, ethosomes have more capability to retain the stability of caffeic acid than SLN. Furthermore, this experiment allows for calculating lag time and apparent diffusion coefficients. Lastly, the reversible gluing or blending of ethosomes and SLN to skin membrane in terms of change in skin resistance has been measured. This strategy is very beneficial to study how these nanosystems interact reversibly with skin and how they change the skin resistance for a short time period without affecting skin integrity [32,119]. The integrity of the skin in terms of skin impedance have been measured by four electrode assembly [119,172].

## 9. Conclusions

Although nanomedicine is still at an initial phase of development, many therapeutic active agents that exploit nanotechnology have been accepted and commercialized. Their clinical translation is still a big question because the physical and in vitro characterization is not capable enough to predict their clinical efficacy. Therefore, several controversies regarding the selection of characterization protocols arise. Moreover, the inherent nature of nanoparticles may cause challenges to their consistent production and application in reproducible studies. Awareness of dynamic instability of suspended nanosystems could eventually be solved or addressed thanks to the knowledge of the chemical characteristics of the system surface. The application of a well-defined and thoughtful characterization plan together with the collection of storage information could be useful tools for addressing reproducibility problems or identifying possible variations.

In this review, we tried to cover several techniques for the physical characterization of nanosystems and the challenges associated with them. By means of this review, illustrating the use of the characterization methods of lipid-based nanosystems together with their advantages and limitations, we also tried to explain how they can be effectively combined and complement each other. It has been demonstrated that the techniques presently available strongly help the comprehension of the structure, the behavior, and the stability of the produced nanosystems. Presenting each technique in a comparative way, this review can be used as a guide, helping researchers in choosing the most suitable technique for their characterization and a precise evaluation of their use. However, the modalities of interaction between the nanosystem matrix and the loaded molecules are still and there are open questions regarding the selectivity, reliability, and reproducibility of each technique. Indeed, researchers are increasingly aware of what needs to be measured. Therefore, for significant progress to be made toward this goal, much more effort is needed to establish testing criteria, validate efficacy, and accumulate safety data. Obviously, the accuracy and resolution of many techniques will need to be further improved in the future, and we hope that this review will also help define which techniques are worth the effort for further technical improvements. It would be better to pay attention to optimizing the best-fitting techniques for achieving new research goals.

## Figures and Tables

**Table 1:** Principal methods of lipid-based nanoparticle production together with their advantages and limitations.

Nano-Carrier	Method of Preparation	Merits	Demerits	References
Liposomes	Ethanol injection	- Simple and safe - Reproducibility - No oxidative degradation of lipid	- Heterogeneous population - Very diluted liposomes - Difficulty in complete ethanol removal	[25,26,27]
	Direct hydration	- Simple - Production speed	- Not transposable on industrial scale - Require further sonication or extrusion	[28,29]
	Reverse phase	- Uniformity of size and Lamellarity - High aqueous space-to-lipid ratio - More embedding of aqueous material	- Denaturation of loaded proteins due to exposure of organic solvents	[27,30]
Ethosomes	Cold Method	- Simple approach - No sophisticated instrumentation required	N/A	[31,32]
	Hot method	- Ehanolic mixture is heated to 40 °C - Useful for both hydrophilic/hydrophobic drugs	N/A	
SLN/ NLC	Hot high-Pressure Homogenization	- Scaling up feasible - No use of organic solvent	- Extremely energy intensive process - Drug degradation owing to high temperature - Limited drug loading	[33]
	Cold high-pressure homogenization	- No drug degradation - Useful to load hydrophilic drugs -No alteration in crystallization	- Larger diameter of particles - Extensive size distributions	[34,35]
	Micro emulsion-based method	- No energy required - Good theoretical stability	- Very much susceptible towards alterations - Low yield of nanoparticles - High dilution of final dispersion	[35,36]
MAD/ Cubosomes	Top-down approach	- No aggregation - Higher encapsulation efficiency	- High energy input - Not applicable for thermo-sensitive moieties - Poor cubosomes quality due to use of high temperature (40 and 60 °C)	[37,38,39]
	Bottom-up approach	- Required less energy inputs - No high temperature requirements - Long term stability - Homogenicity	- Higher dilution used	[37]

**Table 2:** Structural properties of some lipid phases.

Phase	Type	Structure Elements	Class
1D: lamellar	-	Lamellae	-
2D: hexagonal	I or II	Infinitely long rods	Rod-like
3D: Cubic, P4_3_32	II	Rod network and micelles	Mixed rod-like and micellar
3D: Cubic, Pm3n	I	Micelles	Micellar
3D: Cubic, Pn3m	II	Intertwined rod networks	Bicontinuous (IPMS * Diamond-surface)
3D: Cubic, Fd3m	II	Micelles	Micellar
3D: Cubic, Im3m	II	Intertwined rod networks	Bicontinuous (IPMS * Primitive-surface)
3D: Cubic, Ia3d	I or II	Intertwined rod networks	Bicontinuous (IPMS * Gyroid-surface)

* IPMS = infinite periodic minimal surfaces.

**Table 3:** Factors affecting accuracy of DLS measurements.

Factor	Description	References
**Solvent used in sample**	It should be underlined that some solvents (toluene) have tendency to scatter the light up to certain extent, which can create background noise in results. Secondly, dimethyl sulfoxide can alter viscosity of the sample at different temperature conditions	[133,134]
**Concentration of sample**	Higher concentration of sample corresponds to higher number of particles. It means that light hit so many times before reaching to detector and finally loses intensity. Too diluted sample cannot produce scattered light to be analyzed. DLS is only useful in case of diluted samples.	[135,136]
**Agglomeration**	Nanomaterials have tendency to undergo agglomeration. The bigger size of agglomerate scatter light with great extent. Even it can destroy the detector also.	[99]
**Type of cuvette**	Use of organic solvent or temperature conditions higher than 50 °C can interfere with Cuvette made up of plastic. Cuvette should be clean properly with detergent or distilled water.	[103,131]

**Table 4:** Comparison between SdFFF, DLS, and cryo-TEM.

Technique	Principle	References
**SdFFF**	Based on separation of nano/ micro scale particles as a function of their specific mass with known particle density by assuming that particles are spherical. The dimensions illustrate the diameter of an equivalent sphere. It gives higher resolution comparably to PCS because it fractionates different sized particles first more specific for the dispersions having multimodal size distributions.	[110,139]
**PCS/DLS**	It measures the particle diameter by light scattering could give misleading interpretation with systems having non-spherical particles. Moreover, larger spheres monopolize the scattering behavior of the sample, small numbers of large nanoparticles result in a considerable enlargement of size and dispersity of nano-dispersion.	[110,140]
**Cryo-TEM**	It is not possible for all the particles to be imaged because larger particles can be neglected in the analysis. Therefore, usage of cryo-TEM for estimating diameter could give uncertain results because distances or geometries may be over- or under assessed.	[103,110]

**Table 5:** Z-average and zeta potential values of some nanoparticles reported in literature.

Type of Nanoparticle (np)	Active Molecules	Z-Ave (nm) ^1^	ζ Potential (mV) ^2^	Reference
SLN	caffeic acid	201 ± 11	−4.92 ± 0.01	[119]
ethosomes	caffeic acid	219 ± 21	+1.99 ± 2.48	[119]
liposomes	QSi ^3^	230 ± 12	+55.8 ± 0.4	[102]
liposomes	Peptides	200–350	17.8 ±13	[144]
cationic particles	Peptide Nucleic Acids	870–1140	+27.9 ± 4.2	[145]
chitosan	Enoxaparin	135.2 ± 3.1	31.67 ± 4.6	[146]
stearylamine lipid		180.3 ± 3.6	−13.52 ± 2.3	
polymer lipid hybrid	Selegiline	178.7 ± 3.4	−25.07 ± 3.4	[147]
thiolated chitosan		215 ± 34.7	+17.06	

^1^ Zeta average diameter (mean size). ^2^ Zeta Potential. ^3^ Quorum sensing inhibitors.

## Data Availability

Exclude this statement.
