# Correction: Hallan et al. Challenges in the Physical Characterization of Lipid Nanoparticles. *Pharmaceutics* 2021, *13*, 549

**DOI:** 10.3390/pharmaceutics16121583

**Published:** 2024-12-11

**Authors:** Supandeep Singh Hallan, Maddalena Sguizzato, Elisabetta Esposito, Rita Cortesi

**Affiliations:** 1 Department of Chemical, Pharmaceutical and Agricultural Sciences, University of Ferrara, I-44121 Ferrara, Italy; hllsnd@unife.it (S.S.H.); sgzmdl@unife.it (M.S.); ese@unife.it (E.E.); 2 Biotechnology Interuniversity Consortium (C.I.B.), Ferrara Section, University of Ferrara, I-44121 Ferrara, Italy

In the original publication [1], the literature mentioned in Figure 6 was not correctly cited. The correct reference should be reference [73,107,108]. The citation has now been inserted into Section **4. Morphological Characterization of Nanoparticles**, Section *4.1. Cryo-Transmission Electron Microscopy*, and should read:

As shown in Figure 6, it is evident that—notwithstanding what is reported in literature by some researchers indicating that both methods resulted in the production of cubosomes when poloxamer 407 is used as surfactant—in the case of the hydrotrope method, uni- or bi-lamellar vesicles were obtained [73,107,108].

In the original publication [1], there was a mistake in the legend for Figure 6, which was missing the reference to the literature and Elsevier permissions. The correct legend appears below.

**Figure 6**. Cryo-TEM images of MAD obtained with hydrotrope (**a**) or hot homogenization (**b**) method in the presence of poloxamer 407 [73]. (With Elsevier permission, License Number 5785341232886).

The authors state that the scientific conclusions are unaffected. This correction was approved by the Academic Editor. The original publication has also been updated.
